# Current and Emerging Medical Therapies in Pituitary Tumors

**DOI:** 10.3390/jcm11040955

**Published:** 2022-02-12

**Authors:** Nicolas Sahakian, Frédéric Castinetti, Thierry Brue, Thomas Cuny

**Affiliations:** Department of Endocrinology, Hôpital de la Conception, Centre de Référence des Maladies Rares Hypophysaires HYPO, INSERM, U1251, Marseille Medical Genetics, France and AP-HM, Aix-Marseille Université, 13005 Marseille, France; nicolas.sahakian@ap-hm.fr (N.S.); frederic.castinetti@ap-hm.fr (F.C.); thierry.brue@ap-hm.fr (T.B.)

**Keywords:** pituitary tumors, acromegaly, cushing’s disease, prolactinoma, dopamine agonist, somatostatin

## Abstract

Pituitary tumors (PT) represent in, the majority of cases, benign tumors for which surgical treatment still remains, except for prolactin-secreting PT, the first-line therapeutic option. Nonetheless, the role played by medical therapies for the management of such tumors, before or after surgery, has evolved considerably, due in part to the recent development of well-tolerated and highly efficient molecules. In this review, our aim was to present a state-of-the-art of the current medical therapies used in the field of PT and the benefits and caveats for each of them, and further specify their positioning in the therapeutic algorithm of each phenotype. Finally, we discuss the future of PT medical therapies, based on the most recent studies published in this field.

## 1. Introduction

Pituitary adenomas (PA), also referred to, especially in Europe, as pituitary neuroendocrine tumors (PitNETs), or simply as pituitary tumors (PT) [1] represent benign tumors in a majority of cases. Their treatment may involve a combination of surgery, medical therapies (such as Dopamine Agonist (DA) or Somatostatin Receptor Ligand (SRL)), and radiotherapy because of their potentially severe impact on mortality, morbidity, and quality of life of affected patients [2,3]. Transsphenoidal surgery still represents, except for prolactinomas (PRLomas, also named prolactin (PRL)-secreting pituitary tumor), the first-line treatment of PT; however, local invasiveness of the tumor may render surgical resection more difficult, sometimes leading to the use of medical therapies in the first line [4]. Besides the specific cases of aggressive PT, which may require the use of medical therapies from the oncology field (see review [5]), clinicians have to deal with complex issues concerning the conventional treatment of PT, and these are summarized as follows:

▪ Prolactin-secreting PT resistant to dopamine agonist therapy. This situation is often encountered in young men and associated with a higher risk of aggressive tumors [6]. Surgery and radiotherapy are not efficient enough to control the tumor and, ultimately, temozolomide, or even check-point inhibitors, can represent therapeutic options [5,7]. Overcoming resistance-to-Dopamine Agonist (DA) currently represents the most relevant challenge in the medical treatment of prolactin-secreting PT.
▪ Growth hormone secreting PT (GH-secreting PT) that are resistant or insufficiently controlled by first-generation somatostatin receptor ligands (SRLs). Second-line treatments are discussed as well as radiotherapy and/or second surgery.
▪ Adrenocorticotropic hormone secreting PT (ACTH-secreting PT, formerly Cushing’s disease) that are not visualized by pituitary MRI raise the question of whether surgery should be performed in first intention. If not, the choice to begin a steroidogenesis inhibitor and how to titrate it, regarding the induced risk of adrenal insufficiency, implies a close follow-up.
▪ Eventually, non-functioning PT represent one of the most represented phenotypes of PT; however, few medical therapies are available so far. We will discuss potential targets of interest in this review.

## 2. Material and Methods

A comprehensive systematic search was undertaken to identify appropriate primary literature and reviews in PubMed (https://pubmed.ncbi.nlm.nih.gov (accessed on 1 June 2021)), published from 2010 until 2021. All guideline papers for each topic were reviewed. Papers published before 2010 were selected based on their known relevance from the scientific community.

Furthermore, references from relevant papers were analyzed to identify any appropriate literature missed through the systematic search.

Our search was performed using associated keywords “Pituitary adenomas” OR “Acromegaly” OR “Cushing’s disease” OR “Prolactinomas” OR “Thyrotropinomas” OR “Non Functioning Pituitary adenomas” AND “Medical treatment” OR “Medical therapies” OR “Pituitary drug” OR “dopamine agonist” OR “somatostatin analogs” OR “Pegvisomant” OR “Steroidogenesis inhibitors” AND:

- Prolactinoma section: “Prolactinoma” OR “Prolactin-secreting pituitary tumor” OR “Lactotroph tumor” OR “Lactotroph adenoma”;
- Acromegaly section: “Acromegaly” OR “GH-secreting pituitary tumor” OR “Somatotroph tumor” OR “Somatotroph adenoma”;
- Cushing’s disease section: “Cushing’s disease” OR “ACTH-secreting pituitary tumor” OR “Corticotroph tumor” OR “Corticotroph adenoma” OR “Silent Corticotropinomas”;
- Non-Functioning Pituitary Adenoma section: “Non-Functioning Pituitary Adenoma” OR “NFPA” or “Gonadotroph Pituitary Adenoma”;
- Thyrotroph Pituitary Adenomas: “Thyrotroph Pituitary Adenoma” OR “Thyrotropinoma” OR “TSH-secreting Pituitary Tumor” OR “TSH-secreting Pituitary Adenoma”.

Our search identified 686 related articles. We selected 227 papers of potential significance to the literature review.

## 3. Current Medical Therapies Approved in the Treatment of PT

### 3.1. Prolactin-Secreting PT

**Figure 1:**
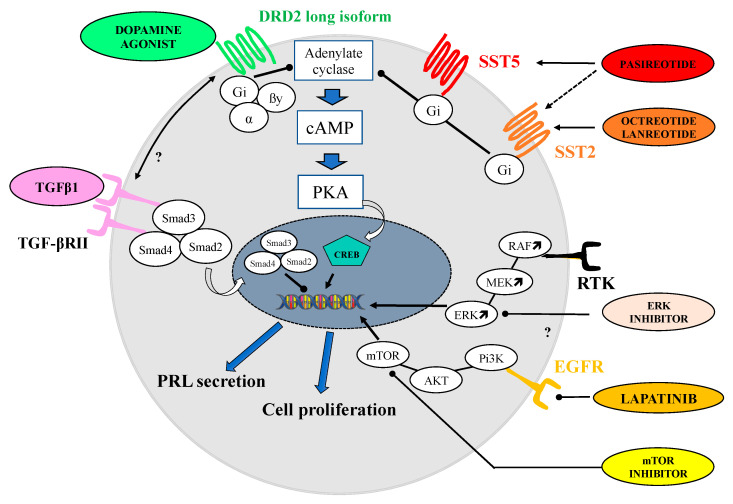
Schematic representation of the main molecular mechanism by which medical therapies (DA and other) act in prolactinoma and signaling pathway involved in the response to dopamine agonist therapy or medical treatment in prolactin-secreting pituitary tumor. For the sake of clarity, major signaling pathways are simplified, and readers can refer to the manuscript for more details. On the right side of the cell, optional therapies and the ones that are in development are represented. The question mark means a possible effect supported by in vitro experiments.

**Figure 2:**
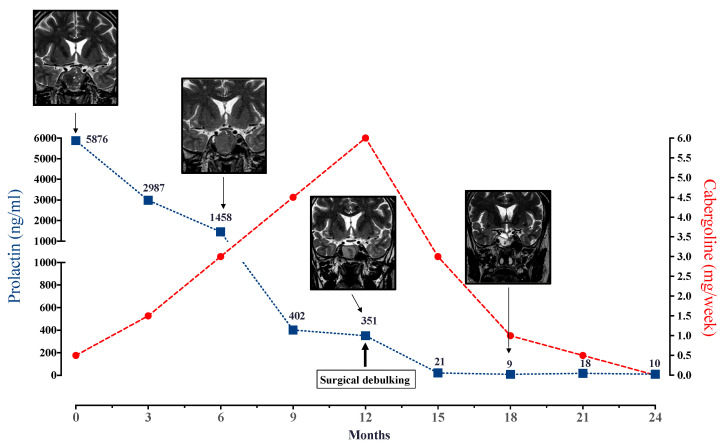
Journey of a male patient diagnosed with invasive to the right cavernous sinus macroprolactinoma. A substantial decrease of prolactin occurred with an increasing dose of cabergoline (+0.5 mg/week every month) up to 6 mg/week during the first year without, however, reaching normalization. A decrease of the tumor volume ≤ 50% also occurred. According to the definition, the patient was resistant to cabergoline. Because the antitumor effect under CAB also occurred in the right invasive part of the tumor, the patient was operated on with success (MRI scan shows fat in the sellar region at month 18), such that a positive outcome was obtained 18 months after initial diagnosis.

Dopamine agonists (DA) represent the cornerstone of the medical treatment of prolactinomas (PRLomas) and, therefore, are always discussed in first intention, either in micro- (tumor diameter < 1 cm), macro- (1–4 cm), or in giant (≥4 cm) PRLomas [8]. As such, DA undoubtedly represent one of the most efficient treatments in the era medicine, because the pharmacological targets of interest in PRLomas are well known. DA bind with high affinity to the dopamine receptor subtype 2 (DRD2), which is intensely expressed in lactotroph and PRLoma cells [9]. Once binded, DA lead to inhibition of PRL secretion through a combination of two recently reviewed mechanisms [10], namely, the inhibition of adenylyl cyclase by G_i_ and/or G_0_ proteins, coupled to DRD2 in the intracellular compartment, which ultimately reduces intracellular concentration of cAMP, and a decrease in intracellular calcium concentration, which occurs by inhibition of the phosphatidylinositol metabolism. The antiproliferative effect exerted by DA is rather due to a simultaneous increase of ERK phosphorylation and beta-arrestin-2 dependent AKT dephosphorylation [10,11]. These molecular mechanisms, by which medical therapies (DA and others) act in prolactinoma, are summarized in Figure 1 (for more detail regarding the molecular mechanism and main signaling pathways involved in the response to dopamine agonist therapy in pituitary lactotrophs tumoral cells, see review [12]). Three DA, namely Bromocriptine, Cabergoline, and Quinagolide, are currently commercialized. Bromocriptine (BRC) represents the historical compound which is, currently, almost exclusively used in cases of intolerance to other DA [13,14]. BRC efficacy, defined on the basis of normalization of PRL and significant tumor shrinkage (≥50% from initial tumor volume), is close to 90% in patients with microPRLomas, and 70% in patients with macroPRLomas [7]. However, side effects, such as nausea, vomiting, symptomatic hypotension, dizziness, and headache, are not rare and are reported to occur in up to 10% of patients [15]. Another limitation of BRC is its short half-life (about 90 min), which imposes a twice- or thrice-daily administration and, subsequently prompted the development of new DA with longer lasting effects and improved side effect profiles, such as cabergoline (CAB). CAB is a selective DRD2 agonist, with a weekly administration [16]. In a randomized multicentre clinical trial, including 459 women with hyperprolactinemic amenorrhea, CAB was more effective than BRC in restoring both normoprolactinemia (83% vs. 59%, respectively, *p* < 0.001) and fertility (72 vs. 52%, *p* < 0.001). Interestingly, CAB also had a better tolerance profile than BRC, with 68% having adverse effects in the CAB group as compared to 78% in the BRC group [17]. In another reference study, CAB normalized PRL in 86% of hyperprolactinemic patients (*n* = 455), with normalization of PRL in 92% of patients with microPRLomas and in 77% of patients with macroPRLomas [18]. The antitumoral effect of CAB occurs in the short term, allowing an improvement of visual field abnormalities in 70 to 90% of patients, with a rapid tumor shrinkage observed in up to 67% of cases [18,19,20]. Consequently, CAB is preferred to endoscopic decompression of the optic chiasm, in the case of macro or giant PRLoma with visual field abnormalities [21]. A recent study showed that tumor shrinkage by the third month predicted long-term response to CAB of patients with macroPRLoma [22]. This also means that, somehow, a subset of patients will be resistant to the action of DA. Resistance to DA is generally defined as failure to normalize PRL levels and to achieve a tumor size reduction of at least 50% from the initial volume at the dose of ≤2 mg/week of CAB [23]. However, this definition has to be regularly questioned, given the fact that a further two thirds of patients with macroPRLomas will achieve a normalization of their PRL levels under CAB, on average in less than two years (Figure 2) [24]. When comparing CAB and BRC in DA-resistant patients, it is noteworthy that CAB further normalized PRL in 70% of BRC-resistant patients [17,25,26,27,28]. The side effects of CAB are similar to those reported for BRC, but are generally less frequent, less severe, and of shorter duration [8]. Regarding long-term therapy, the risk of cardiac valvulopathy justifies the carefully evaluation of heart function and valve morphology before and during treatment with CAB. One consequence is that a vast body of research, including retrospective and prospective case–control series, national database studies, meta-analyses, expert reviews, and guidelines, has been conducted on this topic and point out that CAB, used at the general dose labelled in endocrinology (up to 2 mg/week), does not increase the risk of valvulopathy, even in the case of long-term treatment [29]. Moreover, this should encourage alleviation of the yearly echocardiographic follow-up that is often recommended. In patients treated with a higher dose of CAB (>2 mg/week), or those with subclinical and asymptomatic modifications of valve morphology, it remains necessary to maintain an annual echocardiographic assessment. Few patients have badly experienced the onset of a compulsive behavior (such as excessive gambling and hypersexuality) under CAB [30]; these side effects remain rare, occurring in less than 5% of patients, and warrant a reduction of the posology [31]. Finally, a fear that emerged with ergot-derived compounds, namely ergotism (i.e., a severe vasoconstrictive manifestation due to alkaloid actions), is exceptional [32].

The third DA currently available is quinagolide (QNA), which differs from the others because it is not derived from ergot. It is usually administrated once- or twice-daily in patients with PRLomas, and led, in pioneered studies, to a significant reduction of tumor size and PRL levels in 90% of patients with microPRLomas, and normalization of PRL level in 50% of them [33]. It also allowed normalization of PRL in some patients previously resistant to BRC [34]. Compared to BRC treatment, adverse side effect are less frequent during QNA administration.

### 3.2. GH-Secreting PT (Acromegaly)

Acromegaly is a rare but severe endocrine condition, resulting from growth hormone (GH) secretion by a PT (GHoma) or, in very exceptional cases, by ectopic secretion of growth hormone-releasing hormone (GHRH) [35,36]. When feasible, surgery represents the treatment of choice in acromegaly. Alternatively, first-generation SRLs, octreotide and lanreotide, represent the first-line medical treatment in patients with acromegaly and are usually discussed in three different scenarios: (1) when surgery fails to cure the patient, with a persistent oversecretion of GH, (2) in patients with pronounced acromegalic symptoms and/or comorbidities, therefore at high surgical/anesthesia risk, and (3) in patients who refused surgery [37]. A proper estimation of the efficacy of SRLs to control GH hypersecretion is still lacking due to the absence of randomized, prospective studies, which would include, moreover, a homogeneous group of acromegalic patients. In the past, data from a meta-analysis reported an overall biochemical control rate close to 55% for GH and insulin-like growth-factor 1 (IGF-1) [38]. However, in SRL-naive patients, an efficacy rate close to 40% seems to be more commonly observed [39,40]. The development of SRLs for the treatment of acromegaly has been known for decades [41] and relies on the inhibitory effect exerted by native somatostatin over GH secretion [42]. Somatostatin binds the five different somatostatin receptor subtypes (SST1–5), and SRLs have a high binding affinity for SST2, and, to a lesser extent, for SST5 and SST3. Once bound, SRLs trigger the inhibition of adenylyl cyclase and, subsequently, of cAMP synthesis. By doing so, SRLs contribute to reduced intracellular calcium concentration that, in turn, inhibits GH secretion and somatotroph proliferation [10]. In GHomas, SST2 and SST5 are predominantly expressed followed by SST3, SST1, and SST4 [43]. Using SRLs in acromegalic patients in the preoperative period is routine. However, according to the Endocrine Society clinical practice guidelines, SRL therapy before transsphenoidal surgery is advisable for patients with severe pharyngeal swelling, sleep apnea, or high-output heart failure to reduce surgical risk from severe comorbidities [44]. Noteworthy is that first-line therapy with SRLs can also lead to a significant reduction (i.e., ≥20% of the initial tumor maximum diameter) of the pituitary tumor [45,46,47]. Indeed, in a 48-week multicenter prospective study, 63% of acromegalic patients treated with 120 mg monthly subcutaneous lanreotide experienced a significant reduction of their tumor volume after 4 months [46]. The response to SRL is influenced by clinical and paraclinical parameters: for instance, young male patients with a high level of GH at diagnosis are less responsive to SRLs [48]. At the pathological level, GHomas with high values of Ki67 and sparsely granulated (SG) pattern display a poorer response to SRLs [48], which is associated with a lower density of SST2 at the cell membrane of SG tumors, and a T2-hyperintensity signal on the MRI [49,50]. Recently, Wildemberg et al. showed, in an elegant study, that somatic mutations of the *gsp* oncogene, identified in nearly 40% of GHomas, were not predictive of response to SRLs [51]. Familial predisposition to acromegaly has been known since the end of the 1990s [52], and germline mutations of the *AIP* (Arylhydrocarbon receptor interacting protein) gene are found in approximately 15% of FIPA (Familial Isolated Pituitary Adenoma) syndrome cases [53]. *AIP*-mutated pituitary tumors have clinical features that may negatively impact treatment efficacy, as suggested by the study from Daly et al., which compared 96 *AIP* mutated as compared to 232 *AIP*-negative GHomas. *AIP* mutations were associated with larger, higher GH levels, and more frequent extension to the cavernous sinus as compared to controls and, eventually, *AIP* patients were less sensitive to SRL [54]. In this series, none of the patients from four FIPA kindreds with *AIP*-mutated tumors responded to treatment with octreotide, whereas the acromegalic patients in the *AIP* negative family were controlled with a standard dose of 20 mg monthly of octreotide LAR [55]. In *AIP*-positive GHomas, uncontrolled by surgery, and first generation SRL, pasireotide could be an option [56]. In an in vitro study, resistance to first-generation SRLs was associated with a low degree of *AIP* expression by the tumor, including in *AIP*-negative GHomas [57], suggesting that *AIP* seems to mediate response to SRLs in acromegaly. Conversely, Pasireotide sensitivity was the same, whatever the degree of *AIP* expression (50 vs. 40%; *p* = 0.74). In an observational retrospective study, 77 *AIP*-negative acromegalic patients were screened for both Aryl hydrocarbon Receptor (AHR) rs2066853 variant, and glutathione-S-transferase-P1 (GSTP1) gene promoter methylation [58]. A total of 17 patients carried the AHR rs2066853 variant, and 26 had methylated GSTP1gene promoter. The later were more resistant to SRLs (*p* = 0.02) as compared to GSTP1 unmethylated patients. Patients with non-methylated GSTP1 and AHR wild-type, were the most sensitive to SRL treatment, while those with both the GSTP1-methyl and the AHR rs2066853 variant were all resistant to SSA (*p* = 0.01) [58].

Another recent syndrome predisposing to gigantism and known as X-linked acrogigantism (X-LAG) was described in 2014 [59]. In this syndrome, it was shown that biochemical control of GH oversecretion with SRL was not readily achieved, despite moderate to high levels of expression of SST2 in tumor tissue [60]. Thus, in 18 patients with X-LAG, nine patients underwent first-line medical therapy, and none achieved control of secretion despite the use of therapeutic doses of either octreotide (40 mg monthly) or lanreotide (90 mg monthly). In these patients, surgery was performed in all cases, and secretion control was achieved in three cases but at the cost of hypopituitarism. For the remaining six patients, additional treatment with first-generation SSA still did not achieve control of hypersecretion, which justified the addition of pegvisomant. Overall, repeated surgeries, external radiotherapy, and a combination of medical therapies were required for the control of GH oversecretion in the setting of X-LAG [60].

The current guidelines of acromegaly can be applied to all patients, whatever the genetic background identified. If the biochemical control of the patient is insufficient, it is recommended to either increase SRL dose and/or administration frequency, which can lead to an additional 25% rate of controlled patients [61,62,63], or discuss a second-line medical therapy.

Other labelled medical treatments in acromegaly include Pasireotide (PAS), another SRLs characterized by a high affinity to SST2, SST1, and SST5 [64,65]. PAS showed a superior efficacy compared to octreotide or lanreotide in achieving biochemical control of SRL-naive and SRL-resistant acromegalic patients, in a phase III clinical trial [66]. Since this study, PAS has been labeled for the treatment of acromegaly in second line [37] and can further cause a 25% decrease in tumor volume [66,67]. The main concern related to PAS is the onset of hyperglycaemia-related adverse effects in 40 to 60% of patients [67], of whom 60 to 70%, depending on the dose of PAS, will achieve a goal of HbA1c <7% with Metformin, a biguanide insulin sensitizer drug, alone or in combination with other oral antidiabetic medications [68]. In the remaining patients (i.e., those who are still beyond 7% in spite of metformin), incretin-based therapy is a suitable option for controlling hyperglycaemia events [69].

Dopamine agonists (DA), mainly CAB, represent another therapeutic option in acromegaly [70], but their efficacy to control GH secretion is not related to the degree of DRD2 expression in the tumor cells [71]. As such, no predictor of response to CAB currently exists, except the fact that CAB monotherapy is usually discussed in mildly-secreting GHomas (IGF-1 less than two-fold the upper limit of the age-adjusted normal range) as a first-line or second-line therapy. Its efficacy to normalize IGF-1 is obtained in ~35% of patients [37,72]. In case of partial resistance to SRLs, the addition of CAB to SRLs is an option and can lead to IGF-1 normalization in 50% additional patients [72].

**Figure 3:**
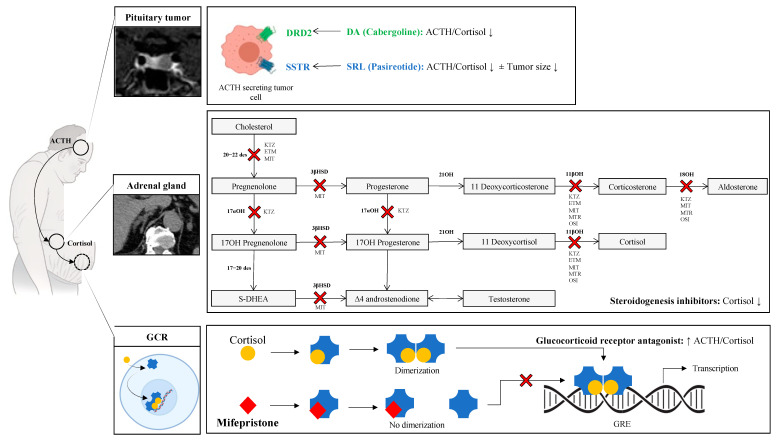
Schematic representation of medical therapies available in the treatment of Cushing disease: 20–22 des = 20–22 desmolase; 17αOH = 17a hydroxylase; 3βHSD = 3b hydroxysteroid deshydrogenase; 21OH = 21 hydroxylase; 18OH = 18 Hydrolase; 17–20 des = 17–20 desmolase; GCR = Glucocorticoid Receptor; DRD2 = Dopamine Receptor subtype 2; SST = Somatostatin Receptors; SRL = Somatostatine Receptor Li gand; DA = Dopamine Agonist; GRE = Glucocorticoid Response Element. Steroidogenesis inhibitors: KTZ = Ketoconazole/Levoketoconazole; ETM = Etomidate and analogs; MIT = Mitotane/op’ddd); MTR = Metyrapone; and OSI = Osilodrostat. ↓ = decrease; ↑ = increase.

Pegvisomant (PEG) represents a GH receptor antagonist developed at the beginning of the 1990s, whose principle is to compete with endogenous GH at the binding site of the GH receptor, and, by so doing, to decrease circulating IGF-1 [73]. The half-life of the molecule was increased through a pegylation process to make it suitable for daily injection given the very short half-life of the endogenous GH. In clinical practice, PEG is indicated as a second-line or third-line therapy, even though some authors recently discussed its relevance in SRL-naive acromegalic patient [74]. PEG is discussed in patients in whom surgery has failed and/or in those who are poor responders to first-line SRLs. It can either be administered in monotherapy or in combination with SRLs. Given alone, PEG led to normalization of IGF-1 in ~90% of patients in clinical trials, which means, in patients carefully followed up with control visits and proper dose adjustment. In reality, the efficacy of PEG to control IGF-1 is rather close to 65% [75]. When combined with SRL, normal IGF-1 levels are achieved in the long term in 97% of acromegalic patients with a weekly dose varying from 30 to 300 mg of PEG [76]. According to the guidelines, the current algorithm for the treatment of acromegaly is depicted in Figure 3. 

### 3.3. ACTH-Secreting PT (Cushing’s Disease)

**Figure 4:**
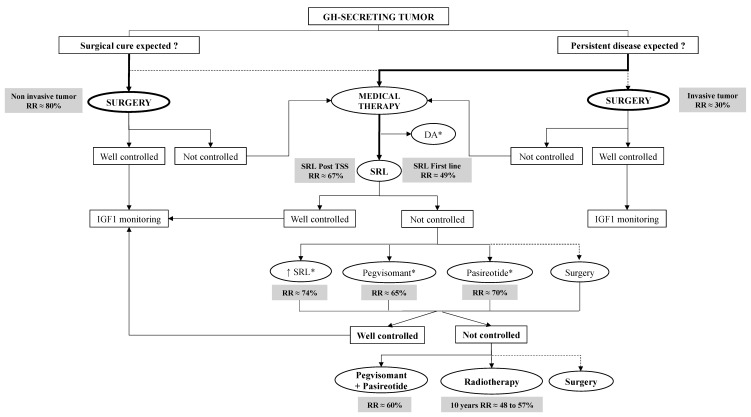
A proposed algorithm for the treatment of acromegaly, considering the reported remission rate at different stages of the management. SRL = Somatostatin Receptor Ligand (Octreotide or Lanreotide); IGF1 = Insuline like Growth Factor 1; RR = Remission Rate/Controlled rate; DA* = consider Dopamine Agonist as Cabergoline in case of mild disease and/or cosecreting GH/PRL secreting tumor; ↑ SRL* = consider increase SRL dose and/or reduce time between injection in case of partial response to SRL; Pegvisomant* = consider Pegvisomant if glucose impairment; Pasireotide* = consider Pasireotide if tumor is of concern.

Cushing’s syndrome (CS) is consecutive to Cushing’s disease (CD) in about 70% of cases, the remaining cases being represented by ectopic ACTH production (10% of cases) and ACTH-independent CS of adrenal origin (20% of all CS) [77]. Surgery represents the treatment of choice for CD; however, it can be preceded by anticortisolic drugs, with the aim of mitigating surgical bleeding and reducing perioperative morbidity (hypertension, diabetes) [78]. Recurrent or persistent CD after surgery represent the main indications of using medical therapies in CD. The latter are usually classified into three categories: pituitary-targeting drugs, steroidogenesis inhibitors (SI), and glucocorticoid receptor (GR) antagonists [78] (Figure 4).

#### 3.3.1. Pituitary-Targeting Drugs

The aim of pituitary-targeting drugs is to inhibit the ACTH secretion of corticotroph adenoma cells. In vitro studies showed that corticotrophs express significant levels of dopamine receptor subtype 2 (DRD2) and somatostatin receptor subtype 5 (SST5) at the membrane [79] and, therefore, CAB and PAS were logically tested in CD patients. In a phase III, randomized, double-blind, clinical trial including 162 patients with CD, subcutaneous PAS normalized urinary free cortisol (UFC) after 6 months in 15% and 26% of patients treated with 600 μg and 900 μg, respectively, twice a day [80]. After 12 months and 24 months of treatment with PAS, 19 and 34% of patients had a normalized UFC, respectively [80,81]. A subset of patients (*n* = 8) of this study had a significant reduction of tumor volume (>25%) after 24 months of treatment with PAS; 7 out of 8 had a corticotroph microadenomas [82]. Following this phase III trial, once-monthly subcutaneous PAS (10 and 30 mg) was tested in CD patients, and UFC normalization occurred in 42% and 41% of the two group, respectively, at month 7 [83], while an extension of this study (Median overall exposure to PAS at study end of 23.9 months) was accompanied by an additional 53% of patients with normalized UFC at last assessment [84]. Nevertheless, 89% of patients were classified as having diabetes at the last assessment of this study.

CAB targets DRD2, which is expressed in 80% of corticotroph tumors [85], and can inhibit ACTH secretion in vitro [86]. In CD patients, few studies assessed the efficacy of CAB to control hypercortisolism and, furthermore, their outcomes are discrepant. A retrospective, multicenter cohort study yielded normal UFC in 40% of patients in the first year, but only 23% of those showed sustained UFC normalization after a median of 32.5 months [87,88]. A prospective study assessing the efficacy of CAB in the short term (6 weeks), did not find any significant antisecretory effect in 20 CD patients [89]. Two potential concerns must be mentioned regarding the use of CAB in CD: first, some CAB-responders had doses of CAB up to 7 mg/wk, a posology clearly higher than the one used in PRLomas, with the potential risk of cardiac valve regurgitation. Second, use of CAB in CD patients was recently associated with a higher risk of recurrence following conventional fractionated radiotherapy [90].

#### 3.3.2. Adrenal-Directed Steroidogenesis Inhibitors

Since initial studies with amphenone B in the 1950s [91], steroidogenesis inhibitors (SI) have been particularly useful for the treatment of CS. In CD, they are employed after failure of pituitary surgery, as a bridge until radiation therapy becomes effective, or as primary therapy in some patients with severe hypercortisolism who have contraindications to surgery [92]. Presently, their availability differs from country to country. Furthermore, variability in study design and quality makes a comparison of drug efficacy difficult to establish. Historical agents used are ketoconazole and metyrapone, and the most recently developed are osilodrostat and levoketoconazole [93].

Ketoconazole is an imidazole derivative with antifungal properties that can be administered orally. In a retrospective study including 200 patients with CD, ketoconazole yielded a normalization of UFC in 33/51 patients (64.7%), treated for at least 24 months with a mean dose of 600 mg per day [94]. A significant improvement of CS symptoms were also noted (blood pressure, glucose metabolism, muscle weakness, and decreased bodyweight). However, approximately 15–20% of CD patients eventually overcame the treatment efficacy [94,95], and mild hepatotoxicity (liver enzymes ≤5 ULN) occurred in another 10–20% of patients in the first 6 months of treatment [96].

Metyrapone is a pharmacologic agent used in the diagnosis of adrenal insufficiency and occasionally in the treatment of CS. The primary inhibitory target is 11ß-hydroxylase and, to a lesser extent, 17-α-, 18-, and 19-hydroxylase (Figure 4). Metyrapone led to normalization of UFC (over a mean follow up of 8.7 months) in 71% of patients, irrespective of the primary etiology (adrenal of pituitary), with up to 18% of patients relapsing after initial response [95]. One retrospective study, including 164 patients with CS, showed that a mean dose of 1040–1465 mg per day of metyrapone resulted in normalization of UFC in 43% of patients over a mean period of 8 months [97]. Likewise, metyrapone administered at a median dose of 1000 mg/day over 9 months led to a marked decrease of UFC (−67%) and late night salivary cortisol (LNSC, −57%) after 1 month of treatment in 31 patients with CS (including 20 with CD) [98]. The main adverse effects reported with metyrapone are hirsutism (which is likely a long-term side effect), hypokalaemia, hypertension, and dizziness. While warranting attention, adrenal insufficiency is rare, observed in up to 10% of patients.

Osilodrostat is an 11ß-hydroxylase and aldosterone synthase inhibitor more recently developed that has shown safety and efficacy for reducing cortisol secretion in CD patients in phase II studies [99,100]. Thereafter, 137 patients with CD were enrolled in a phase III, double-blind, prospective, multicenter, study, LINC 3 [101], of which 72 (53%) entered the randomization process after 24 weeks. Amongst them, 31/36 (86%) from the osilodrostat arm maintained normal UFC as compared to 10/34 (29%) in the placebo arm (OR 13.7, 95% CI 3.7–53.4, *p* < 0.0001). In the extension phase of the LINC 2 study, most of the patients (≈80%) were controlled in the long term (up to 6 years) with osilodrostat [102]. The biochemical control under osilodrostat was associated with a substantial improvement of clinical symptoms in patients, such as a decrease in bodyweight, blood pressure, and cholesterol fractions. A better quality-of-life and depression scores were also noted [101]. A subsequent phase III, double-blind study comparing titrated osilodrostat versus placebo in patients with persistent CD or de novo non-candidate to surgery CD, was designed (LINC 4). By week 36, 80.8% (CI 69.9–89.1) of patients had normalization of their UFC [103]. The main adverse effects observed with osilodrostat are less than grade 3, with nausea, anemia, and headaches in approximately 10% of patients. While a subset of patients will be required to take glucocorticoid replacement, the incidence of adrenal insufficiency requiring the interruption of the drug remains low: 2 to 3% [103,104]. An excess of adrenal steroid precursors can be encountered under osilodrostat, such as hypokalemia, hypertension, or even hirsutism, seen in 10% of women [103].

The last SI, and the more recently developed, is a 2S, 4R enantiomer of ketoconazole, named levoketoconazole. This compound has a conformational configuration that confers to this molecule similar hypocortisolic properties (by blocking the same steroidogenic enzymes) with, however, in theory, less hepatic toxicity (by a weaker inhibitory effect on hepatic CYP7A) [105].

A phase III clinical trial, single-arm study tested twice-daily administration of levoketoconazole in 94 patients with CS, among which, 80 had CD. By end-of-dose titration, 81% of patients had mUFC normalization [105], with the most common adverse events being nausea (32%) and headache (28%). The drug is still under investigation and not approved by either the FDA or the EMA.

Mitotane targets 20,22-desmolase, 11β-hydroxylase, and 18-hydroxylase enzymes. It is characterized by its long-lasting adrenolytic action, its potentially irreversibility, and its slow onset of action and high pharmacodynamic variability. It does not represent a treatment of choice in CD. Etomidate is an intravenously administered anaesthetic drug, possessing strong anticortisolic properties [106]. Its use is reserved for patients hospitalized in intensive care units with severe hypercortisolism and therefore is not recommended for the treatment of CD. Chemical modifications of etomidate have been under preclinical studies in order to reduce the sedative-hypnotic activity while maintaining its adrenocortical steroid synthesis inhibition activity [107,108]. The glucocorticoid receptor (GR) blocker Mifepristone is currently the only glucocorticoid and progesterone receptor antagonist available and is FDA-approved for the treatment of non-surgical CS with metabolic complications (hypertension and/or glucose intolerance). A previous study enrolling 43 patients with CS showed a significant improvement of blood pressure and glucose profile in 38% and 54% of patients, respectively [109]. Overall, 87% of patients had a significant improvement in clinical status. Its pharmacological action makes the monitoring of ACTH and cortisol in patients uninformative and is, therefore, evaluated on the improvement of clinical symptoms.

### 3.4. TSH-Secreting PT (TSHomas)

Despite the recently described significant increase in their incidence and prevalence [110], thyrotropin-secreting pituitary neuroendocrine tumors (TSHomas) represent the rarest subtype of PT, accounting for 0.5 to 2% of all PT [110]. They represent a rare cause of central hyperthyroidism characterized by normal or inappropriately high Thyroid-stimulating hormone (TSH) concentrations in the presence of elevated free-thyroid hormones (fT3 and fT4) [111]. The treatment of choice for TSHomas is surgery, with a complete resection of the tumor achieved in two-thirds of patients [112]. The rather disappointing results of surgery (especially for the frequently detected macroadenomas) indicate that additional treatment, such as radiotherapy or medical treatment, may be necessary. Medical therapy with SRLs can be considered, in a case-by-case approach, as they effectively reduce TSH secretion in approximately 80% of patients and induce shrinkage in about 45% of TSHomas. Molecular predictors of response to SRL remains largely unknown; however, Gatto et al. showed that a high SST5/SST2 ratio at the cellular membrane of TSHoma cells was associated, in vitro, with a better response to SRLs [113]. A dramatic response to SRLs was also observed: even thyreotroph deficiency could occur in a subset of TSHomas patients treated with SRLs (after a median time of 4 weeks) [114]. DRD2 is inconsistently expressed on TSHoma cells [9], which is the reason why treatment with DA results in heterogeneous and often poor responses, with the best results reported in mixed TSH/PRL-secreting adenomas [115]. In some cases, it should be noted that adding DA to SRLs resulted in a sustained control of both biochemical parameters and tumor growth [116].

### 3.5. Non-Functioning PT

Non-functioning pituitary tumors (NFPT) are largely represented by gonadotroph tumors, such as silent ACTH; GH or PRL adenomas should not be included in this class of pituitary tumors [117]. Transsphenoidal surgery is the first option in NFPT, eventually combined with external radiotherapy [118]. However, a high expression level at the membrane of DRD2 was noted in such tumors [119]. DA treatment results in tumor shrinkage in a non-negligible 30% of cases [120], potentially also due to the local inhibition of VEGF (vascular endothelial growth factor) release [121]. Recently, a single-center, open-label, 2-years randomized clinical trial showed that CAB, used for the treatment of residual NFPT, was associated with a higher progression-free survival rate as compared to controls (*p* = 0.01), independently of the DRD2 expression in the tumor [122]. This study, therefore, reactivates the debate of using CAB for treating residue of NFPT rather than as a first-line therapy [123]. Unlike GHomas, SST2 is sparsely expressed in NFPT [124], and the correlated efficacy of SRL therapy is therefore moderate, with tumor shrinkage in less than 10% of cases [125]. Finally, the multi-receptor ligand PAS was tested in NFPT and showed promising antiproliferative effects in vitro on NFPT primary cultures [126], although it never demonstrated equivalent results in the clinical setting. Finally, TBR-760, a chimeric compound that acts on both DRD2 and SST2 was able to inhibit proliferation of NFPT cells [127] and showed similar results in aggressive NFPT developed in mice [128]; however, this effect was not superior to those obtained with CAB.

## 4. New and Alternative Therapies for the Treatment of PT

### 4.1. Prolactin-Secreting Pituitary Adenomas

In DA-resistant PRLomas, surgical debulking and/or radiotherapy are usually not sufficient to control hyperPRL and only lead to 50% normalization or less [129,130]. Currently, there is no large clinical trial on innovative medical treatment in DA-resistant prolactinomas [12]. Alternative medical therapies are, therefore, actively discussed in a patient-individualized approach.

#### 4.1.1. Switching to Another Dopamine Agonist

The option of switching from CAB to another DA is unlikely to be efficient in these circumstances, despite exceptional published clinical cases suggesting possible efficacy of BRC [131].

#### 4.1.2. Somatostatin Receptors Ligands

Although SST2 is expressed in PRLomas, SRLs are generally inefficient to inhibit PRL secretion and/or reduce tumor volume [132]. Some studies noticed that SRL, in addition to DA, could further improve the control of PRL secretion [133,134]. Likewise, a few reports suggest that PAS could be a valuable therapeutic option in DA-resistant prolactinomas, in particular when a significant expression of SST5 is found in the tumor [135,136,137].

#### 4.1.3. Metformin

Metformin, a biguanide, is a diabetes drug that activates AMP-activated protein kinase (APMK) and is suggested to have antitumoral properties. Interestingly, it has been reported that activation of AMPK subsequently inhibits mammalian target of rapamycin (mTOR), which, in turn, would induce autophagy-dependent cell death in PRLomas [138]. In preclinical studies, metformin used in both DA-sensitive and -resistant cell lines (MMQ and GH3, respectively), in combination with bromocriptine, showed a greater reduction of PRL-secretion and diminished better xenografts growth, compared to bromocriptine alone [139]. Other studies already found a reduction in cell viability and an increase in apoptosis of pituitary tumor cell lines (GH3 cell lines) through AMPK-dependent and independent mechanisms [140]. Two published clinical cases showed that metformin in combination with BRC resulted in normalization of PRL secretion, together with a subsequent decrease of tumor volume, after 24 months of treatment [141]. In a recent pilot study, including ten DA-resistant PRLomas under CAB treatment (2–7 mg/week) for at least 6 months, metformin was introduced (dose from 1 to 2.5 g/day, according to patient tolerance). CAB dose was unchanged during metformin therapy. No patient reached PRL normalization, but two of them were considered partial responders for exhibiting a PRL reduction >50% at single time point during metformin [142]. Further studies are required to assess the potential benefit of metformin in larger cohorts of DA-resistant PRLomas.

#### 4.1.4. Inhibition of EGF Receptor

The epidermal growth factor (EGF) system is a family of transmembrane tyrosine kinase receptors known as EGFR, ErB1, or HER that, when binding ligands, forms homo and/or heterodimers and transduces intracellular signaling through intrinsic kinase domains [143]. In human PRLomas, EGFR expression has been shown to be heterogeneous [144,145,146]. In preclinical models, gefitinib, an EGFR antagonist decreased cell proliferation, blocked PRL gene expression and decreased both volume and secretion levels of PRLomas rodents xenografts [147]. Some reports suggested that EGFR inhibition in human PRLomas that are resistant to DA and/or are aggressive may be an effective therapeutic option [145].

#### 4.1.5. TGF-Beta Signaling Pathway

Transforming Growth Factor (TGF) is another well-known cytokine with several roles, including the regulation of lactotroph proliferation as well as their hormonal secretion [148]. The TGFb signaling cascade is triggered by the binding of either TGFb1, TGFb2, or TGFb3 to the type II TGFb receptor, which, in turn, recruits and phosphorylates the type I TGFb receptor to form a complex [149]. Subsequently, type I TGFb receptor leads to the formation of a heterodimeric complex between smad2, smad3, and smad4, which, ultimately, translocates into the nucleus to regulate expression of various genes [150]. Sarkal et al. previously showed that TGFb1 inhibits both PRL secretion and proliferation of lactotrophs, in a manner similar to dopamine [151]. Additionally, they showed that transformed PRL-secreting PR1 cells have lost the TGFb1 growth inhibitory response, while still having conserved the TGFb1 PRL-mRNA inhibitory response [152]. In an elegant study, Recouvreux et al. demonstrated that TGFb1 secretion and type II TGFb receptor expression were both decreased following the administration of dopamine antagonist or after DRD2 knockdown. These results suggested that TGFb1 is involved, at least in part, in mediating dopamine functions on lactotrophs cells, namely the inhibition of PRL secretion and the control of lactotroph proliferation [153]. In the setting of DA-resistant PRLomas, a significant downregulation of the TGFb/smad signaling pathway was observed as compared to normal human pituitaries. In GH3 cells, TGFb1 and the oestrogen antagonist fulvestrant led to significant dose and time-dependent cytotoxicity. Moreover, GH3 cells treated with fulvestrant showed increased active TGFb1 levels and a decreased PRL secretion [154]. Diminished TGFb1 activity and decreased expression of the TGFb1 system downstream effectors have been described in animal models of PRLomas as well as in human ones [148]. These features suggest that recovering local TGFb1 activity could be an interesting way to management DA-resistant PRLomas.

#### 4.1.6. MAPK Kinases and Pi3K/AKT/mTOR Signaling Pathways

The RAF/MEK/ERK and PI3K/AKT/mTOR signaling pathways are crucially involved in cell growth and metabolism, but also in the tumorigenesis process [155,156]. In pituitary neuroendocrine tumors, AKT, which is at the crossroads of the mTOR pathway, is overexpressed and overactivated, although the consequences of this high activity remain to be elucidated [157]. Indeed, downstream AKT, the level of phosphorylated forms of targeted effectors, such as mTOR, TSC2, or p70S6K, was found to be comparable between normal pituitary and pituitary tumor samples [158]. On the other hand, the expression of the phosphorylated forms of MEK1/2 and ERK1/2, the downstream effectors of the MAPK signaling pathway, was significantly higher in PRLomas as compared to normal pituitaries [158]. Interestingly, it is likely that an intricate crosstalk between the ERK and PI3K signaling pathways occurs in the murine cell line GH4T2, in which PI3K would appear as a counterregulatory mediator of the ERK-induced PRL transcription [159]. In the same cell line, the inhibitory effect obtained with CAB over PRL secretion and cell proliferation was mediated through the phosphorylation of S6K, a target of mTOR; therefore, it is likely involved in the response to DA in PRLomas. Besides the response to DA, the mTOR signaling pathway was recently identified as a promotor of pituitary tumor development in PRLomas [160]. As a matter of fact, in vitro experiments suggest that rapamycin, a mTOR inhibitor, could be effective in the treatment of PRL tumoral cells [161]. Until now, there is only one published case of a DA-resistant patient who experienced a significant decrease of both PRL levels and tumor volume after 5 months of treatment with everolimus, a mTOR inhibitor [162]. Moreover, the immunohistochemical analysis of the PRLoma tumor in this patient revealed a high level of p-AKT, p4EBP1, and p70S6K [162]. While this case illustrates a potential interest of everolimus in DA-resistant prolactinomas, there is still a lack of randomized, phase III clinical studies to support this therapeutic strategy [162].

### 4.2. GH-Secreting Pituitary Adenomas

#### 4.2.1. New Formulations of Somatostatin Receptor Ligands

##### Oral Octreotide

Currently available parenteral SRLs achieve, in most cases, biochemical control and symptomatic improvement in acromegaly. However, these uncomfortable routes of administration cause challenges to patient in terms of treatment adherence, injection-related burden, and quality of life improvement; these are some of the reasons why oral SRLs have been under development over the last few decades. Considering the limited passive intestinal absorption of protein or peptides, lipidic formulation were considered for SRL oral formulation [163]. Oral octreotide acetate is formulated with oily suspension (Octreotide/OS), filled into hard-shell gelatin capsules, then enteric coated to create a stomach resistant capsule [163]. This formulation allows a transitory and reversible epithelial opening of the tight junctions between intestinal epithelial cells, and ultimately paracellular absorption of the drug [163]. Moreover, current data suggest that the Octreotide/OS-induced permeation may prevent molecules larger than 70 KDa, thus, limiting the risk of the internalization of immunoglobulins and intestinal pathogens [163]. In preclinical studies, no signs of toxicity were observed in monkeys during and following daily administration of octreotide/OS for 9-months, and no treatment-related mortality was recorded [163].

In a phase I study [164], oral octreotide with single dose from 3 to 20 mg and injectable octreotide at a dose of 100 µg were assessed in 75 healthy volunteers. Both oral and injectable octreotide were well tolerated. Both 20 mg oral octreotide and 100 µg subcutaneous octreotide showed comparable pharmacokinetic profiles. Oral administration of 20 mg octreotide acetate suppressed the basal GH levels by 49% (*p* < 0.05) and the secretory response to GHRH stimulation by 80% (*p* < 0.001) [164]. In a subsequent phase III study including 155 fully or partially controlled patients with acromegaly (i.e., IGF-1 < 1.3ULN and 2 h integrated GH < 2.5 ng/mL) treated with injectable SRL for more than 3 months, were switched to dose escalation (from 40 to 80 mg/d) oral octreotide with the purpose to control IGF-1 for at least 7 months [165]. Overall, 98 (65%) and 93 (62%) of all enrolled subjects were responders up to 7 and 13 months, respectively, as compared to 134 (89%) at the baseline visit while on injectable SRL. Oral octreotide also showed efficacy in maintaining clinical response. Most commonly side effects were mild to moderate and included gastrointestinal, neurological and musculoskeletal disorders [165].

Moreover, 56 biochemically controlled patients with injectable SRL treatment were randomized between oral octreotide and placebo treatment. Compared to placebo group, oral octreotide treatment maintained IGF-1 levels within normal limit between the baseline and the end of study (Oral octreotide: Mean IGF-1 0.80 ULN and 0.97 ULN, respectively; Placebo: Mean IGF-1 0.84 ULN and 1.69 ULN, respectively). In the oral octreotide group, 16 (58%) patients met biochemical response (IGF-1 < 1 ULN) at the end of 36 weeks period compared to 5 (19%) in the placebo group (*p* = 0.008 OR 5.77 95% CI 1.44–28.21). Mainly reported sides effect were gastrointestinal disorders (diarrhea, nausea, vomiting, abdominal discomfort) and all were mild to moderate [166]. Oral octreotide demonstrated consistent results for persistent biochemical response with comparable safety profile than injectable SRL [167].

##### Octreotide Subcutaneous (SC) Depot

Octreotide SC depot is a novel, ready-to-use octreotide formulation that has been developed in order to overcome the limitations of the long action release (LAR) formulation. In a phase I study including 122 healthy volunteers randomized between monthly octreotide SC depot and octreotide LAR, octreotide SC depot showed a four- to five-fold greater bioavailability as compared to octreotide LAR and a more rapid and extensive suppression of IGF-1 after the first injection compared with octreotide LAR. Considering the safety profile, octreotide SC depot was well tolerated, and the most adverse effects were gastrointestinal impairment, mild to moderate in intensity [168]. In a phase II study including five adults patients with acromegaly previously treated with octreotide LAR, pharmacokinetics analysis of octreotide SC depot showed a significantly higher maximal concentration and AUC during the first 28 days, as compared to octreotide LAR. Three patients had IGF-1 levels lower than ULN at the end of the pre-randomization period and maintained their IGF-1 levels up to the end of the treatment period. Moreover, four had GH levels < 2.5 ng/mL at all time points during the study. Octreotide SC depot was well tolerated, and the observed adverse effects were consistent with the previously known safety profile of octreotide with grade 1–2 gastrointestinal disorders, injection site, pain and headaches [169].

##### Nasal Octreotide (DP1038)

Nasal octreotide has been under development since the 1990s to try to overcome the injectable burden with a noninjectable delivery system. In a preliminary crossover study, nine acromegalic patients were treated with both injectable octreotide at 100 µg and intranasal octreotide, with doses varying from 500 to 2000 µg. Results showed that both injectable and intranasal octreotide rapidly suppressed GH levels with very little differences between the doses [170]. In another randomized trial, intranasal octreotide at a dose of 2000 µg twice daily induced adequate GH suppression in acromegalic patients and was as effective as 100 µg injectable octreotide given four times daily [171]. In recent years, a new intranasal octreotide formulation, named DP1038, was developed. DP1038 is a short-acting noninvasive intranasal formulation of octreotide acetate that includes 1-*O*-*N*-dodecyl-β-d-maltopyranoside excipient for enhanced intranasal absorption. In healthy volunteers, DP1038 showed PK characteristics comparable to injectable octreotide and reached known therapeutic range drug concentrations. DP1068 strongly suppressed GHRH/Arginine stimulated GH secretion, in a range comparable with injectable octreotide. Of note, intranasal octreotide was well tolerated and did not show significant safety findings [172].

#### 4.2.2. New Drugs Targeting GH Axis

New drugs are currently being developed, and some of them have entered phase I/II clinical trials. They are extensively reviewed in these recent works [65,173]. Among them, paltusotine, formerly CRN00808, is an oral nonpeptide small SST2 agonist. In healthy volunteers, 10 mg of paltusotine resulted in a 91% suppression of GHRH-stimulated GH, and maximal IGF-1 suppression was achieved for 10 days. The safety profile was consistent with somatostatin agonist activity, and the adverse effects were all mild and transient [174]. Veldoreotide, also known as COR-005/Somatropim, is an SRL with an affinity for SST2, SST4, and SST5 that showed, in rat models, high selective somatostatinergic activity and subsequent in vivo inhibition of GH secretion, with minimal or no effect on glucagon and insulin release [175]. In primary cultures of human fetal pituitaries and GHomas, somatoprim suppressed GH secretion by 54% and 35%, respectively. In three out of eight GHomas, somatoprim was more potent than octreotide in suppressing GH release [176]. Octreotide and somatoprim were compared in 27 GHomas cultures. For both drugs, GH suppression was above 80% after six hours in six cell cultures. Interestingly, six octreotide-resistant tumors significantly responded to somatoprim [177]. A phase II study showed, in 20 untreated acromegalic patients, that somatoprim (dose from 100 to 1800 µg) led to a similar inhibition of GH secretion as compared to octreotide (300 µg) [178]. Finally, ONO-5788 and ONO-ST-468 represent two new orally available SST2 agonists that have shown promising in vitro and in vivo results [65,179,180]. Antisense oligonucleotides that target GHR mRNA (ATL1103 and ISIS 766720, respectively) are currently under phase I/II trials [173]. In a phase II randomized open labelled study, Trainer et al. assessed the change in IGF1 at 14 weeks in 26 patients with active acromegaly (IGF1 > 1.3 ULN) after ATL1103 therapy. At week 14, there was a mean IGF1 decrease of 27.8% compared to baseline, with a greater effect in the group with two injections weekly [181].

#### 4.2.3. Dopastatin

Dopastatin defines chimeric somatostatin-dopamine molecules, showing a dual activity over both somatostatin and dopamine receptors. Studies have suggested that these chimeric compounds may facilitate physical interactions between dopamine and somatostatin receptors, resulting in the enhanced activity of both receptors, offering new potential for a variety of diseases with unmet needs, including acromegaly. Somatostatin receptors and DRD2 belong to the same G-protein coupled receptor (GPCR) family, and it has been shown that heterodimerization at the membrane between these two subtypes of receptors can occur [182,183]. The latter is a rare condition at the basal state; however, it strongly increases in the presence of binding ligands, which, in turn, lead to modified agonist binding, enhancement of transduction pathways activation, and increased receptor turnover [182]. The first dopastatin tested was the BIM-23A387 that highly binds DRD2 and SST2 [184]. BIM-23A387 was assessed in 11 primary GHoma cultures from acromegalic patients and resulted in an inhibition of GH secretion in 81% and 39% of fully or partially responders to octreotide, respectively. Moreover, BIM-23A387 was stronger than a combination of DRD2 and SST2 agonists, particularly at low concentrations [184]. BIM-23A760 (or TBR-760) and BIM-23A761 represent the subsequent generation of dopastatin developed. Again, BIM-23A760 and BIM-23A761 led to a maximal GH suppression effect higher than the one observed with octreotide [185]; however, the development of BIM-23A760 in acromegalic patients stopped when evidences showed that its former metabolite, as produced in vivo after repeated injection, had a 200-fold higher dopaminergic activity than the parent compound, and therefore competed with it [186]. As a consequence, a new redesigned dopastatin was developed, TBR-065, that showed a higher effect on GH suppression than TBR-760 (61% vs. 41%). Of note, the metabolite of TBR-065 did not exhibit a notable DRD2 agonist activity [187]. The study of Vazquez-Borrego et al. included nine GHomas in primary cultures and showed that TBR-065 increased apoptosis and the inhibition of GH secretion; the latter effect was even more pronounced that the one observed with either octreotide or pasireotide [188]. Our group recently showed in a series of 17 GH-or GH/PRL-secreting pituitary tumors in primary cultures, that TBR-065 effectively inhibited GH secretion, and further demonstrated that the GH inhibitory effect obtained with TBR-065 was greater than the one observed with either TBR-760 (57 ± 56% vs. 41. ± 12.5%, *p* < 0.001) or octreotide and cabergoline in combination (56.8 ± 7.2% vs. 44.4 ± 9.4%, *p* < 0.001) [189]. In rat models, a significant decrease of the GHoma size after 4 weeks with this dopastatin was also noted [190]. In parallel, TBR-065 was evaluated in humans in a randomized double-blinded trial including 63 healthy men with placebo (*n* = 16), single dose (*n* = 29), or increasing dose (*n* = 18) of injectable TBR-065, with doses up to 2.0 mg daily. A decrease in GH secretion, as well as GHRH-stimulated GH secretion, was observed after 8 and 13 days of treatment. IGF-1 showed a significant change from baseline up to −18 ± 10.1% from the placebo at the dose of 1.0 mg twice daily. Adverse effects, such as orthostatic hypotension and injection site reactions, occurred mainly at a high dose [191].

### 4.3. ACTH-Secreting PT (Cushing’s Disease)

A vast body of research has been conducted for the identification of novel medical therapies in Cushing’s disease (CD) [78,192]. Current available therapies for CD that are proposed preoperatively aim to control hypercortisolism by inhibition of steroidogenesis, even though there is still a matter of debate as to whether prior surgery has to be performed if pituitary MRI does not visualize the lesion [193,194]. Several molecules have been tested to target the corticotroph cells.

#### 4.3.1. Retinoic Acid

Retinoid acid has been identified in previous experimental studies as an inhibitor of both ACTH and cortisol secretion, and corticotroph growth [195] but also as a negative modulator of cortisol secretion at the adrenal level [196,197]. At the molecular level, it was shown that retinoic acid exerted its effect, at least partially, by increasing DRD2 expression in the AtT20 cells, and was associated with sthe ynergistic effect of bromocriptine in nearly 45% of CD primary cultures [198]. Retinoic acid was tested in two prospective clinical trials. The first one resulted in a significant decrease of urinary free cortisol (UFC) (≥50% of baseline value) in 5 out of 7 patients with CD treated with retinoic acid (10 to 80 mg/day) over a period of 6–12 months [199]. Moreover, three out of these five patients experienced normalization of their UFC. The second study included 16 CD patients, uncured by surgery and, subsequently treated by isotretinoin orally for 6–12 months [200]. The results showed a normalization of UFC in six patients and relapse in two of them.

#### 4.3.2. EGFR and BRAF Inhibitors

The inhibition of the EGF receptor (EGFR) signaling pathway represents another therapeutic alternative of interest in CD. Its rationale is a consequence of deciphering the molecular aspects of CD, overwhelmingly represented by somatic mutations of the deubiquitinase gene *USP8* (ubiquitin-specific protease 8) in 35% of tumors [201]. *USP8* mutations were associated with overexpression of EGFR and SST5 in CD [202,203], and EGFR signaling has been recently proposed as a pivotal pathway for corticotroph tumorigenesis [204]. However, directed EGFR inhibitors in CD have not been tested in phase II or III clinical trials until now. In experimental models, gefitinib and lapatinib, showed an antisecretory effect over ACTH release in mice and AtT20 cell lines, respectively [205,206]. The Heat Shock protein 90 (HSP-90) appears also as another critical regulator of the EGFR signaling pathway in CD [207], HSP90 being usually overexpressed in these tumors [208]. Silibinin is an antioxidant drug and a HSP90 C-terminal inhibitor previously used to treat amatoxin poisoning and has been extensively studied as a potential therapeutic of various malignancies. HSP90, a chaperon protein involved in the correct folding of glucocorticoids receptor (GR), is overexpressed in corticotroph adenoma cells [208]. Interestingly, in an allograft mouse model, silibinin demonstrated anti-tumorigenic effects, partially reverted hormonal alterations, and alleviated symptoms of CD [208]. These results suggest that the pathogenesis of Cushing disease, caused by the overexpression of HSP and consequently misregulated GR sensitivity, may be overcome pharmacologically with an appropriate HSP90 inhibitor [208]. Recently, the molecular mechanisms of glucocorticoid resistance in ACTHomas have been extensively reviewed [209,210]. Accordingly, targeting the activity of GR or the factors modulating it, such as HSP90, could represent a valid option for the medical management of CD. Silibinin releases GR from HSP90 and re-established glucocorticoid mediated negative feedback on ACTH secretion. While preliminary studies showed that inhibition of HSP90 decreased both ACTH oversecretion and corticotroph growth [207,208], no clinical studies are available in CD patients.

Another molecular approach of interest is that *BRAF*V600E mutations have been identified in approximately 19% of patients with CD, when *USP8* is non mutated [211]. In that setting, an available *BRAF*V600E inhibitor, verumafenib, led to the inhibition of ACTH secretion in primary cultures of CD [211]. While its use is not labeled for the treatment of CD, this finding could pave the way for a systematic screening of *BRAF*V600E by immunohistochemistry in corticotroph tumors that are non-mutated for *USP8*.

#### 4.3.3. CDK Inhibitor

The role played by cyclin-dependent kinases (CDK) in the tumorigenesis of CD is not completely elucidated to date; however, experimental studies, using the zebrafish model, showed that R-roscovitine (seliciclib), an inhibitor of the cyclin-dependent kinase cyclin E, effectively decreased corticotroph cell growth. Similar results were observed in nude mice xenografted with AtT20 cells, in which R-roscovitine inhibited tumor growth, corticosterone secretion, and ACTH expression [212]. Consequently, R-roscovitine (400 mg orally twice daily for 4 days every week for a total of 4 weeks) entered an interventional phase II clinical study in 4 CD women with baseline mean UFC of 233 mcg/24 h. At the end of 4 weeks of treatment, the mean serum cortisol did not differ significantly (25.6 ± 5.5 at baseline vs. 27.1 ± 8.7 at the end of the study) [213].

#### 4.3.4. New Compounds in Cushing’s Disease

Other molecular targets (over)expressed by the corticotroph cells are currently under investigations for testing new medical therapies. Among them are the arginine-vasopressin receptor AVPR1b, whose antagonism can lead to a significant reduction of ACTH secretion [214]; epigenetic drugs, such as the histone deacetylase inhibitor SAHA, whose effect over the glucocorticoid receptor (GR) transcriptional activity could mitigate the consequence of hypercortisolism [215]; or the nuclear testicular orphan receptor 4 (NTR4), known to stimulate pro-opiomelanocortin expression, ACTH secretion, and growth of experimental corticotroph tumors [216]. Finally, besides pituitary-directed medical therapies, a new GR antagonist, named relacorilant, is also currently being investigated. Its particularity lies in the fact that it does bind, unlike mifepristone, to progesterone receptors. In a phase I clinical study, relacorilant was well tolerated and prevented the effects of the GR agonist prednisone. A phase III, international, double-blind, placebo-controlled, randomized withdrawal study is currently recruiting patients with endogenous CS and concurrent type 2 diabetes mellitus/impaired glucose tolerance and/or uncontrolled hypertension [217].

### 4.4. Non-Functioning and TSH-Secreting PT

There are currently few medical therapies available for the treatment of non-functioning pituitary tumors (NFPT), which contrast with the high representation of this subtype among newly diagnosed PT [218]. NFPT arising from the gonadotroph lineage are characterized by predominant expression of SST3 over the other subtypes of SST, even though the SST expression profile of the tumor was not associated with its aggressivity [219]. In an elegant study, Vàzquez-Borrego et al. tested a new SST3 peptide agonist, over the growth of NFPT [220]. They showed that SST3 agonist could significantly reduce the cellular viability of NFPT primary cultures and further demonstrated that responsive tumors were the ones that expressed the highest levels (both at the mRNA and protein levels) of SST3. Additionally, by silencing SST3, they observed an increase in cellular viability of certain NFPT. Overall, these results suggest that targeting SST3 could be a promising therapeutic option for NFPT uncontrolled by surgery and/or radiotherapy, especially in cases of tumor recurrence. In that setting, assessing the degree of SST3 expression by IHC in the tumor sample may help to distinguish tumors that are likely responders from the others. Currently, there is no clinical study ongoing with the SST3 agonist in NFPT. The same group previously showed that BIM-23A760 (or TBR-760) (100 nM), the SST2/DRD2 chimeric compound, decreased the cellular viability of NFPT in vitro [221]. Whether the new dopastatin TBR-065 would result in similar effects remains to be studied. Eventually, new avenues are currently emerging regarding the immune landscape that surrounds the primitive niche of NFPT [222,223], and, along this line, it seems that medical therapies that act against immune cells such as macrophages or T-lymphocytes could represent targets of choice to control the progression of NFPT.

In the field of TSHomas, there are no new medical therapies currently under investigation. However, it should be noted that a single case report described a complete biochemical response in a TSHoma patient treated with pasireotide as first-line treatment [224].

## 5. Summarizing Conclusions

Along with progress made in the neurosurgical techniques used for the resection of PT in recent years, there was a substantially increased interest for medical therapies developed for the control of tumor growth and/or hormonal hypersecretion. These treatments result from a better understanding of the molecular characteristics underlining tumor mechanisms of secretion and proliferation, with the ambition to offer the patient with chronic disease, the best care with less adverse effects or constraints in the long-term. As such, new approaches are under investigation to overcome the resistance to dopamine agonists in prolactinomas, occurring in approximately 5 to 10% of patients. In acromegaly, oral SST2 agonists represent a real step forward for the patient’s comfort, which may soon lead some of these forms to partly substitute injectable formulations. The medical treatment of hypercortisolism has been recently enriched by the development of new steroidogenesis inhibitors, with fewer adverse effects compared to the ones historically used. Currently, intense investigations are being conducted for the treatment of ACTH-secreting PT. Because non-functioning PT are rather invasive macroadenomas, a body of research is currently focusing on the mechanisms involved in the tumor microenvironment of such tumors. As such, immune cellular components, either surrounding or infiltrating the tumor niche, appear as a target of choice for better comprehending the tumor behavior. More than ever, gathering expert teams for basic and clinical research on the topic of PT, and guaranteeing multidisciplinary management and the best care to patients with PT, represents the prerequisite for optimal management in expert pituitary centers.

## Abbreviations

PA	Pituitary adenomas
PT	Pituitary tumors
PitNETs	Pituitary neuroendocrine tumors
DA	Dopamine Agonist
SRL	Somatostatin Receptor Ligand
PRL	Prolactin
DRD2	Dopamine receptor subtype 2
BRC	Bromocriptine
CAB	Cabergoline
QNA	Quinagolide
GH	growth hormone
GHRH	Growth Hormone releasing hormone
IGF-1	Insulin-like growth-factor 1
SST (1–5)	Somatostatin receptor (subtypes 1–5)
SG	Sparsely granulated
*AIP*	Arylhydrocarbon receptor interacting protein
FIPA	Familial Isolated Pituitary Adenoma
X-LAG	X-linked acrogigantism
PAS	Pasireotide
PEG	Pegvisomant
CS	Cushing’s syndrome
CD	Cushing’s disease
ACTH	Adrenocorticotropic hormone
NFPT	Non-functioning pituitary tumor
SI	Steroidogenesis inhibitors
GR	Glucocorticoid receptor
UFC	Urinary free cortisol
LNSC	Late night salivary cortisol
VEGF	vascular endothelial growth factor
APMK	AMP-activated protein kinase
mTOR	Mammalian target of rapamycin
EGF	Epidermal growth factor
TGF	Transforming Growth Factor
LAR	Long action release
GPCR	G-protein coupled receptor
*USP*8	Ubiquitin-specific protease 8
HSP-90	Heat Shock protein 90
CDK	Cyclin-dependent kinases
NTR4	Nuclear testicular orphan receptor 4
